# Transcranial ultrasonography in the detection of cerebrovascular accident: a systematic review and bivariate random-effects meta-analysis

**DOI:** 10.3389/fneur.2026.1726046

**Published:** 2026-02-19

**Authors:** Ricardo Pinto-Villalba, Andrea Paz, Jordy Arellano, Galo García, Mateo Carrera, Sergio Cardona, Jose A. Rodas, Jose E. Leon-Rojas

**Affiliations:** 1 NeurALL Research Group, Quito, Ecuador; 2 Facultad de Ciencias de la Salud Eugenio Espejo, Carrera de Atención Prehospitalaria y Emergencias, Universidad UTE, Quito, Ecuador; 3 Escuela de Medicina, Universidad Internacional del Ecuador, Quito, Ecuador; 4 Escuela de Medicina, Universidad de Las Américas, Quito, Ecuador; 5 School of Psychology, University College Dublin, Dublin, Ireland; 6 Escuela de Psicología, Universidad Espíritu Santo, Samborondón, Ecuador; 7 Grupo de Investigación Bienestar, Salud y Sociedad, Escuela de Psicología y Educación, Universidad de Las Américas, Quito, Ecuador

**Keywords:** bi-variate meta-analysis, diagnostic power, Doppler ultrasound, stroke, transcranial ultrasound

## Abstract

**Background/objectives:**

Stroke remains a leading cause of disability and death worldwide, with rapid diagnosis critical for effective treatment. Transcranial ultrasonography offers a potentially valuable diagnostic tool, particularly in resource-limited settings. This study aimed to evaluate the diagnostic accuracy of transcranial Doppler (TCD) and transcranial color-coded duplex sonography (TCCS) for detecting ischemic and hemorrhagic stroke.

**Methods:**

We conducted a systematic review and bivariate random-effects meta-analysis following PRISMA guidelines (PROSPERO CRD42023471425). Three databases (Scopus, PubMed, Web of Science) were searched from inception to November 20, 2022. We included studies assessing adult (≥18 years) stroke patients using TCD or TCCS compared to reference standards (MRI, CT, or angiography). Exclusion criteria included pediatric populations, animal studies, vasospasm assessments, and studies with <10 participants. Four reviewers independently screened studies, extracted data, and assessed risk of bias using NIH tools. Primary outcomes were pooled sensitivity and specificity analyzed using bivariate random-effects models; this technique was chosen as it jointly analyses sensitivity and specificity using a random-effects model that accounts for their correlation and between-study variability.

**Results:**

From 16,397 records, 26 studies met inclusion criteria, with 13 studies (*n* = 802 ultrasound examinations) included in meta-analysis. Overall diagnostic accuracy showed sensitivity of 81.1% (95% CI 73.1–87.2%) and specificity of 85.5% (95% CI 71.4–93.3%), with an AUC of 0.874. TCCS demonstrated higher sensitivity for hemorrhagic stroke (87.8%) than for ischemic stroke (77.2%). TCCS showed sensitivity of 74.8% (95% CI 66.4–81.6%) and specificity of 85.2% (95% CI 63.0–95.1%), with an AUC of 0.796. Risk of bias was low in 30.6% of studies, moderate in 50%, and high in 19.4%. Heterogeneity was low to moderate (*I*^2^ = 6.2–23.1%).

**Conclusion:**

Transcranial ultrasonography demonstrates good diagnostic accuracy for stroke detection. While operator-dependent and limited by acoustic window availability, these techniques, after thorough training and validation, may be prioritized in low-resource setting were computed tomography is not readily available through the means of formal capacitation and feasibility studies. The moderate risk of bias in half of included studies suggests a lack of higher-quality research.

**Systematic review registration:**

CRD42023471425.

## 1 Introduction

Stroke, or cerebrovascular accident (CVA), remains one of the leading causes of mortality and long-term disability worldwide, representing a major global public health challenge (1, 2). According to international epidemiological data, stroke accounts for approximately one in six deaths globally and is a primary contributor to years lived with disability, with a particularly disproportionate burden in low- and middle-income countries (LMICs) (1, 3). In many regions, especially in Latin America, Africa, and parts of Asia, stroke incidence and mortality continue to rise, driven by demographic ageing, vascular risk factors, and limited access to specialized stroke care (3, 4). These epidemiological realities underscore the urgent need for rapid and accurate diagnostic strategies, as timely stroke identification and subtype classification are critical determinants of therapeutic eligibility and clinical outcomes (4).

Neuroimaging is central to acute stroke evaluation, as treatment decisions depend on early differentiation between ischemic and hemorrhagic stroke (2, 4). Non-contrast computed tomography (CT) and magnetic resonance imaging (MRI) remain the reference standards for stroke diagnosis and characterization, offering high diagnostic accuracy and anatomical detail (4). However, access to these modalities is frequently constrained by cost, infrastructure, availability, and logistical delays, particularly in prehospital environments, rural hospitals, and resource-limited healthcare systems (5). Even when imaging devices are present, delays related to patient transport, scanner availability, or staffing may hinder timely diagnosis, thereby limiting access to reperfusion therapies and contributing to poorer outcomes (5). This persistent imaging gap has prompted increasing interest in portable, bedside, and lower-cost neuroimaging alternatives that could support early stroke recognition and triage.

Transcranial ultrasonography encompasses a group of non-invasive techniques that allow real-time assessment of intracranial structures and cerebral hemodynamics through acoustic windows in the skull. Among these, transcranial Doppler ultrasonography (TCD) and transcranial color-coded duplex sonography (TCCS) represent distinct but complementary modalities with different diagnostic capabilities (6, 7). Doppler ultrasound is based on the Doppler effect, whereby changes in the frequency of reflected ultrasound waves are proportional to the velocity and direction of moving red blood cells within cerebral vessels. In the context of stroke, Doppler-based techniques enable real-time assessment of cerebral hemodynamics, providing indirect evidence of arterial occlusion, stenosis, collateral flow, or altered intracranial resistance. These hemodynamic measurements form the physiological basis for the use of transcranial ultrasonography in acute and subacute cerebrovascular assessment. TCD primarily provides spectral Doppler information, enabling indirect assessment of cerebral blood flow velocity, flow direction, and pulsatility within major intracranial arteries (6). Its diagnostic value in ischemic stroke lies mainly in the detection of large-vessel occlusion, flow asymmetries, collateral circulation, and microembolic signals, which have been associated with acute ischemic injury and diffusion-weighted MRI abnormalities (6, 7). However, TCD does not provide direct anatomical or parenchymal visualization, limiting its ability to identify intracerebral hemorrhage or mass effect. In contrast, TCCS combines Doppler flow analysis with B-mode and color-coded imaging, allowing partial visualization of brain parenchyma, ventricular structures, and intracranial vessels. This additional anatomical component enables TCCS to detect features such as intracerebral hematomas, intraventricular hemorrhage, midline shift, ventricular enlargement, and parenchymal echogenic changes, while simultaneously assessing cerebral blood flow. As a result, TCCS may offer advantages over TCD in the evaluation of hemorrhagic stroke and space-occupying lesions, although it remains limited by acoustic window availability and operator dependency (6). These conceptual and practical differences between TCD and TCCS are critical when interpreting their diagnostic performance and clinical applicability, particularly across different stroke subtypes.

Although transcranial ultrasonography does not replace CT or MRI where these modalities are readily available, it may serve as a valuable adjunct or interim diagnostic tool in time-sensitive or resource-constrained settings. Previous studies have explored the role of TCD in acute ischemic stroke diagnosis and monitoring, demonstrating reasonable concordance with CT angiography for large-vessel occlusion detection and hemodynamic assessment (6, 7). However, the diagnostic performance of transcranial ultrasonographic techniques has not been systematically compared using bivariate random-effects meta-analytic methods that jointly account for sensitivity, specificity, and between-study variability, limiting a comprehensive understanding of their relative accuracy. Therefore, the aim of our systematic review and bivariate random-effects meta-analysis was to evaluate the diagnostic accuracy of transcranial ultrasonography, specifically TCD and TCCS, for the detection of ischemic and hemorrhagic stroke, using CT, MRI, or angiography as reference standards. By distinguishing between ultrasound modalities and stroke subtypes, our study seeks to clarify their respective diagnostic roles and potential utility, particularly in settings where access to advanced neuroimaging is limited or delayed.

## 2 Materials and methods

### 2.1 Protocol and registration

This study was conducted following the Preferred Reporting Items for Systematic Reviews and Meta-Analyses (PRISMA) 2020 (8) guidelines and its protocol, including the search strategy, was uploaded and registered in PROSPERO (CRD42023471425).

### 2.2 Eligibility criteria

Eligibility criteria was established following the PICO framework (Table 1). The language of the included studies was limited to English and Spanish; other languages were excluded manually during the filtering process. All available studies from the inception of the databases to 20 November 2022 were assessed for eligibility.

**Table 1:** Eligibility criteria.

	Inclusion	Exclusion
Patient		
	Adult, (≥18 years old) with suspected or confirmed ischemic or hemorrhagic stroke in prehospital or in-hospital settings.	Pediatric population (age <18 old), animal models, or other neurological or psychiatric conditions.
Intervention		
	Transcranial ultrasonography, transcranial Doppler ultrasound (TCD), transcranial color-coded duplex sonography (TCCS).	TCD or TCCS used to diagnose vasospasm after hemorrhagic stroke, Moyamoya disease, measure the degree of stenosis, predict risk or determine flow velocity.
Comparison		
	Magnetic resonance (MR), digital subtraction angiography (DSA), computed tomography (CT) to diagnose stroke type.	No applicable
Outcome		
	Diagnostic value of transcranial ultrasonography in the identification of stroke in terms of sensitivity, specificity, positive predictive value, negative predictive value. Studies reporting the frequency of findings in transcranial ultrasonography for ischemic or hemorrhagic stroke	
Study design		
	Original studies	Literature reviews Studies with less than 10 participants Letters to the editor Animal or biomechanical studies

We considered studies using CT angiography (CTA) when it was used alongside non-contrast CT or MRI; CTA served primarily to confirm stroke etiology or to exclude vascular mimics such as dissection or aneurysm, rather than to classify the stroke type (ischemic vs. hemorrhagic). For the purposes of this review, such studies were included as long as a reference standard for stroke classification was present, and CTA was not the sole modality used for determining stroke subtype. This approach reflects the clinical reality in which multiple imaging modalities may be used complementarily during acute stroke evaluation.

### 2.3 Information sources

Studies were identified in Scopus, PubMed, and the Web of Science (WOS); the references of selected articles were also screened. No filters were used.

### 2.4 Search

The full search strategy is available in Supplementary material. The following key terms, with variations, were used: cerebrovascular accident, ischemic stroke, hemorrhagic stroke, transcranial ultrasonography, transcranial Doppler ultrasound, transcranial color-coded duplex sonography, and diagnosis, identification, or screening.

### 2.5 Study selection

Four blinded reviewers carried out an independent and standardized evaluation of the selected studies using Mendeley Reference Manager and Rayyan (9). Deduplication was performed automatically using Mendeley Reference Manager and two stages of screening were performed. During the first stage, reviewers screened titles and abstracts, while the second stage involved full-text review; after these, data of the selected studies was extracted in a spreadsheet. Any discrepancies between the reviewers were resolved by discussion and mutual consensus.

### 2.6 Data items and collection process

For data extraction, we developed a spreadsheet with the following information: author, year of publication, type of study, number of participants, age, type of stroke (ischemic and hemorrhagic), type of transcranial ultrasonography (transcranial Doppler and transcranial color-coded duplex sonography), insonation frequency, sensitivity (true positives and false negatives), specificity (true negatives and false positives), positive predictive value, negative predictive value, standard of reference (magnetic resonance imaging, computed tomography, angiography), main diagnostic signs, and diagnostic criteria. To be included in our meta-analysis, a study had to report both sensitivity and specificity; or provide enough information to determine true positives (TP), false negatives (FN), false positives (FP), and true negatives (TN). In several included studies, diagnostic accuracy was reported based on the number of ultrasound examinations rather than the number of individual patients. A single participant could undergo multiple ultrasound assessments targeting different vascular structures (e.g., bilateral MCA, PCA, or transorbital windows). Due to the aggregated nature of the data in these studies, it was not possible to reliably determine which examinations corresponded to which participants. Therefore, in alignment with the original studies’ reporting practices, we used the number of ultrasound examinations as the unit of analysis when calculating sensitivity, specificity, and other diagnostic metrics. This approach allowed for consistent data extraction across studies, while maintaining the integrity of the diagnostic accuracy estimates. Studies providing partial information (i.e., only reporting sensitivity or specificity in isolation) were excluded from our meta-analysis. If data was missing or inconsistent, we attempted to contact the authors; if they did not answer after repeated attempts, we considered it lost data.

### 2.7 Risk of bias in individual studies

All reviewers assessed the risk of bias independently using the Study Quality Assessment Tools from the National Institutes of Health (NIH) (10). Every question was graded with 1 point, except for questions 1, 2, 3, and 14 for Observational Cohort and Cross-sectional studies and questions 1, 3, 7, 8 for Controlled Intervention Studies which were worth 0.5 points allowing all studies being graded over 12 points. The risk of bias was determined depending on the score obtained in each article, from 0 to 5 as high risk, 6–8 as moderately low risk and from 9 to 12 as minimally low risk. This methodology has been used previously in other systematic reviews (71–73).

### 2.8 Effect measures and synthesis methods

All analyses were conducted in R (11) using the mada package (12). Data was analyzed in a combination of univariate and bivariate methodologies. The univariate analyses included forest plots for both sensitivity and specificity calculated from the reported true positives, true negatives, false positives, and false negatives. A continuity correction of 0.5 was applied to all data to avoid statistical artefacts (13). The bivariate technique maintains the original data’s two-dimensional characteristics. The analysis involves simultaneously examining pairs of sensitivity and specificity, taking into account any correlation that may exist between these two measures using a random effects model with a restricted maximum likelihood estimation; furthermore, this approach allows for and considers between-study variability (14).

Several key metrics were considered to evaluate the model. First, fixed-effects coefficients for sensitivity and false positive rate (FPR) are tested for statistical significance, including both point estimates and a 95% confidence interval. The area under the curve (AUC) provides an overall measure of diagnostic accuracy, with higher values indicating better performance: values of 0.7–0.8 suggest acceptable accuracy, 0.8–0.9 indicate good accuracy, and values above 0.9 reflect excellent accuracy (15, 16). An AUC of 0.5 implies no diagnostic ability, equivalent to random chance. Additionally, heterogeneity (I^2^ estimates) assesses the variability in sensitivity and specificity across studies, which helps determine if observed differences are due to real variations between studies or sampling error. Finally, Akaike Information Criterion (AIC) and Bayesian Information Criterion (BIC) fit indices allow for model comparison, where lower values generally indicate a better fit.

Summary receiver operating characteristic (SROC) plots were also created using the parameters produced by applying the hierarchical summary receiver operating characteristic (HSROC) model. All of the aforementioned calculations were performed irrespective of the type of stroke (i.e., hemorrhagic and ischemic strokes together) for an overall assessment; we also performed subgroup analysis looking into ischemic and hemorrhagic stroke by themselves and stratified by the type of ultrasonographic technique used (TCD or TCCS).

## 3 Results

**Figure 1:**
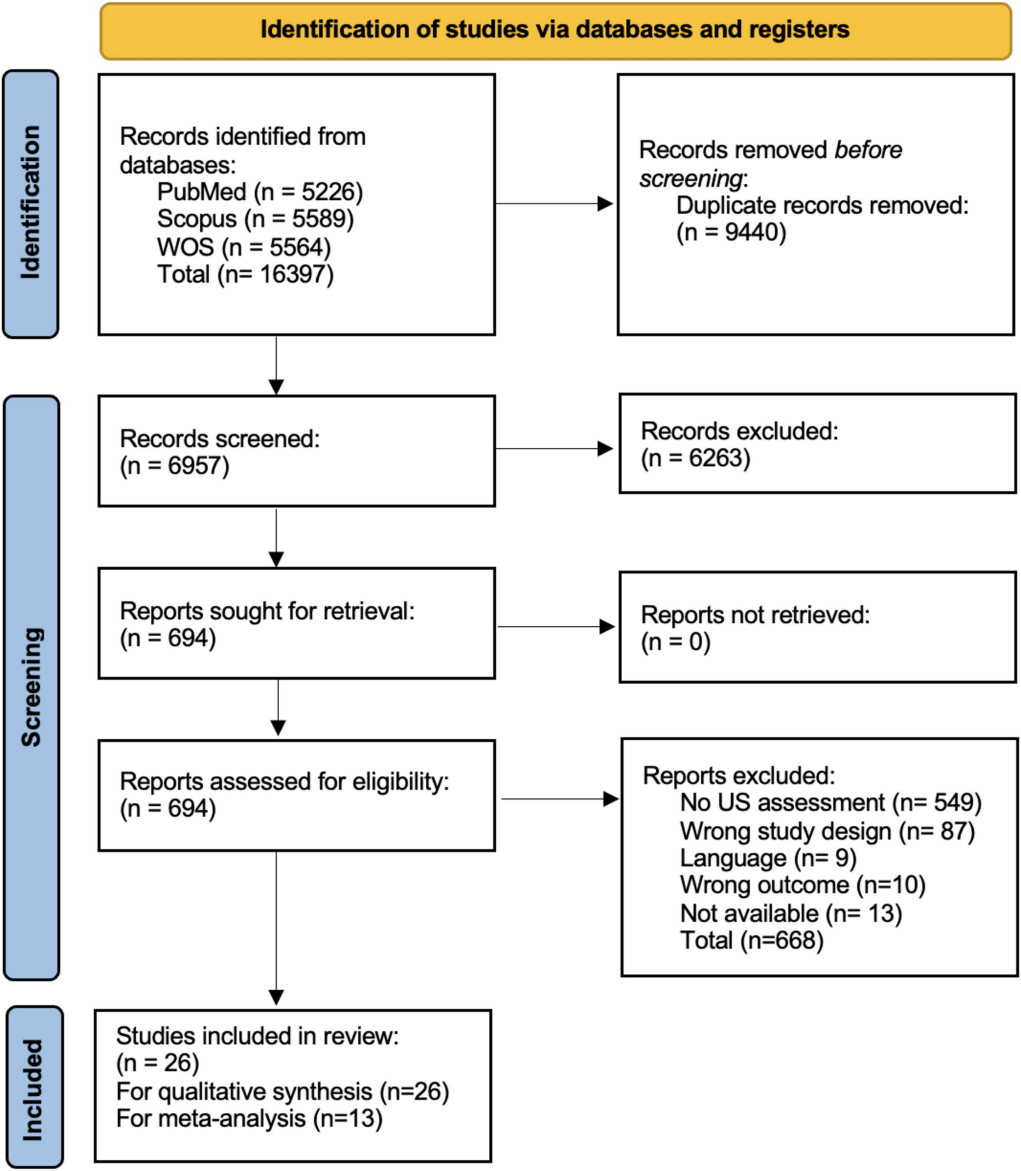
PRISMA flowchart showcasing the selection process.

Our search strategy yielded a total of 16,397 results. Following the removal of duplicate entries, a subset of 6,939 articles remained for further evaluation of which, after the application of our eligibility criteria, 36 articles were finally included (6, 7, 17–50). The complete process of article screening can be found in Figure 1.

The risk of bias was assessed with the Study Quality Assessment Tools from the National Institutes of Health (NIH); from 26 articles included, 9 (34.6%) had minimally low risk of bias, 11 (42.3%) presented a moderately low risk, and 6 (23.1%) had a high risk of bias. Bias assessment of each study is presented in Table 2.

**Table 2:** Risk of bias.

Study	Year	Study design	Risk of bias
Camerlingo et al. (23)	1993	Cross-sectional	Moderately low risk
Martin et al. (30)	1995	Cross-sectional	Moderately low risk
Miralles et al. (35)	1995	Cross-sectional	Moderately low risk
Seidel et al. (44)	1995	Case series	Minimally low risk
Kenton et al. (26)	1997	Cross-sectional	Minimally low risk
Mäurer et al. (31)	1998	Cross-sectional	Moderately low risk
Postert et al. (36)	1998	Case series	Minimally low risk
Alexandrov et al. (17)	1999	Cross-sectional	Moderately low risk
Postert et al. (37)	1999	Cross-sectional	Minimally low risk
Razumovsky et al. (39)	1999	Cross-sectional	Minimally low risk
Lund et al. (27)	2000	Cross-sectional	High risk
Mead et al. (33)	2000	Cross-sectional	High risk
Palomeras et al. (49)	2001	Cross-sectional	High risk
Perren et al. (45)	2004	Cross-sectional	High risk
Marti-Fabregas et al. (34)	2005	Cross-sectional	Moderately low risk
Malferrari et al. (29)	2007	Cohort study	Moderately low risk
Nakajima et al. (7)	2007	Cross-sectional	Moderately low risk
Rathakrishnan et al. (38)	2008	Cross-sectional	Moderately low risk
Madkour et al. (28)	2009	Cross-sectional	High risk
Bar et al. (22)	2010	Cross-sectional	Minimally low risk
Matsumoto et al. (32)	2011	Cross-sectional	Moderately low risk
Schlachetzki et al. (43)	2012	Cross-sectional	Minimally low risk
Herzberg et al. (25)	2014	Cross-sectional	Moderately low risk
Müller et al. (46)	2014	Cross-sectional	High risk
Antipova et al. (18)	2020	Cross-sectional	Minimally low risk
Sarwal et al. (42)	2022	Cross-sectional	Minimally low risk

**Figure 2:**
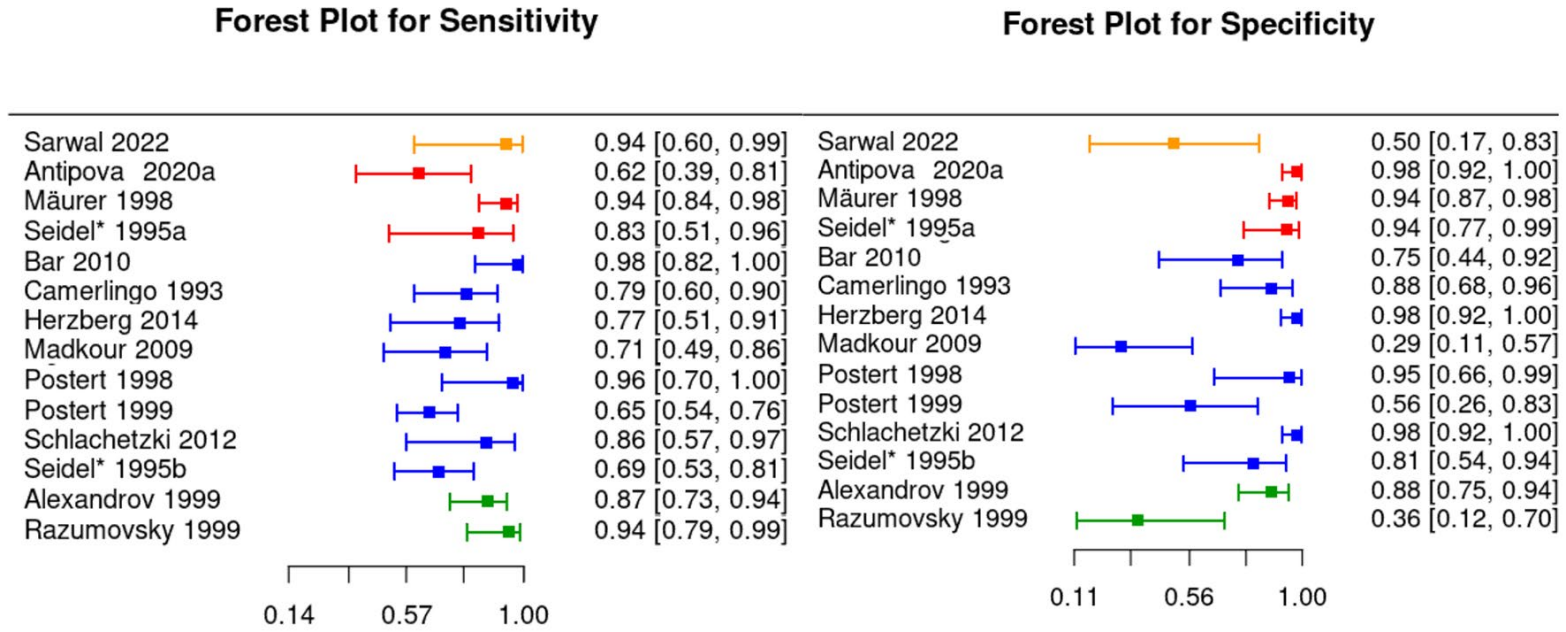
Forest plot showing the values and 95% confidence intervals of sensitivity and specificity. The study in orange looks into hemorrhagic stroke using point-of-care ultrasound; the studies in red look into hemorrhagic stroke using transcranial color-coded duplex sonography (TCCS); the studies in blue look into ischemic stroke using transcranial color-coded duplex sonography (TCCS); and the studies in green look into ischemic stroke using transcranial Doppler ultrasound (TCD).

Thirteen out of the 26 included articles had sufficient information to perform our proposed meta-analysis (17, 18, 22, 23, 25, 28, 31, 36, 37, 39, 42–44). From these, one articles looked into both ischemic and hemorrhagic stroke (44), 9 looked only into ischemic stroke (17, 22, 23, 25, 28, 36, 37, 39, 43), and 2 looked only into hemorrhagic stroke (18, 31, 42). The remaining 13 articles will be included when discussing the pooled frequency of ultrasound findings in stroke patients. Table 3 contains relevant information of the articles included in our meta-analysis, including diagnostic accuracy, type of stroke, and type of ultrasound. Figure 2 contain the forest plots for sensitivity and specificity, organized by type of stroke and ultrasound modality.

**Table 3:** Studies included in the bivariate random-effects meta-analysis and diagnostic accuracy information.

Authors	Year	TP	FN	FP	TN	N US	Sens**	Spec**	Type of stroke	Type of US
Camerlingo et al. (23)	1993	20	5	2	18	45	0.79	0.88	Ischemic	TCCS
Seidel et al. (44)*	1995	7	1	1	22	31	0.83	0.94	Haemorrhagic	TCCS
Seidel et al. (44)*	1995	25	11	2	10	48	0.69	0.81	Ischemic	TCCS
Mäurer et al. (31)	1998	50	3	4	76	133	0.94	0.94	Haemorrhagic	TCCS
Postert et al. (36)	1998	11	0	0	9	20	0.96	0.95	Ischemic	TCCS
Alexandrov et al. (17)	1999	35	5	5	39	84	0.87	0.88	Ischemic	TCD
Postert et al. (37)	1999	44	23	3	4	74	0.65	0.56	Ischemic	TCCS
Razumovsky et al. (39)	1999	25	1	4	2	32	0.94	0.36	Ischemic	TCD
Madkour et al. (28)	2009	13	5	8	3	29	0.71	0.29	Ischemic	TCCS
Bar et al. (22)	2010	22	0	2	7	31	0.98	0.75	Ischemic	TCCS
Schlachetzki et al. (43)	2012	9	1	1	75	86	0.86	0.98	Ischemic	TCCS
Herzberg et al. (25)	2014	11	3	1	71	86	0.77	0.98	Ischemic	TCCS
Antipova et al. (18)	2020	10	6	1	75	92	0.62	0.98	Haemorrhagic	TCCS
Sarwal et al. (42)	2022	7	0	2	2	11	0.94	0.5	Haemorrhagic	POCUS B MODE

FN, false negative; FP, false positives; NUS, total number of ultrasound examinations; POCUS, point-of-care ultrasound; Sens, sensitivity; Spec, specificity; TN, true negatives; TP, true positives; US, ultrasound. *The study provided information for both ischemic and hemorrhagic stroke which was extracted separately. **Sensitivity and specificity values have been calculated using a continuity correction.

### 3.1 Overall analysis

It is important to note that all pooled diagnostic accuracy estimates should be interpreted as conditional summaries within modality- and stroke-specific strata rather than as performance metrics of a single homogeneous diagnostic test.

**Figure 3:**
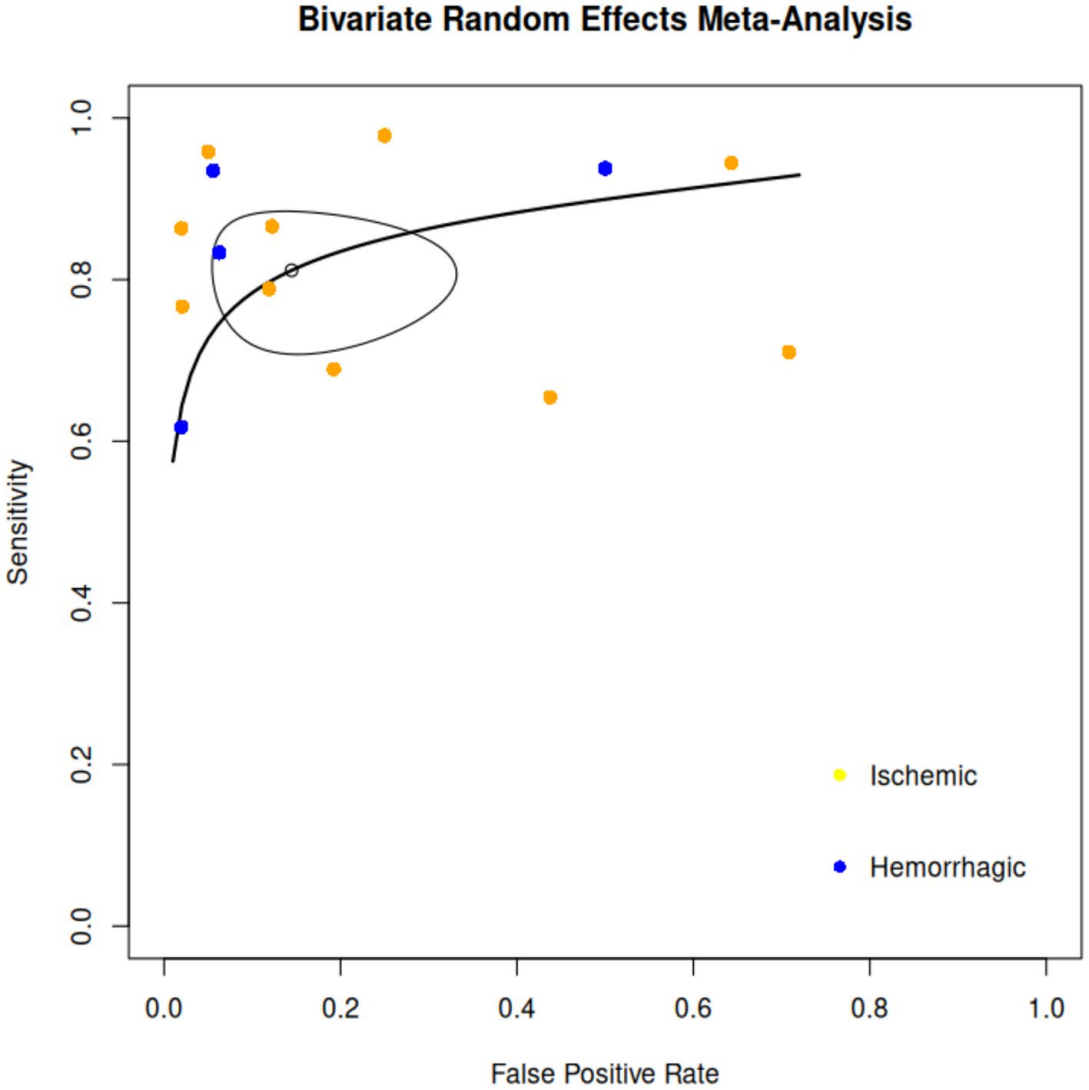
Hierarchical summary receiver operating characteristic (HSROC) curve, showcasing the 95% confidence and predictive regions. Individual study data points are represented in yellow for ischemic stroke and in blue for hemorrhagic stroke.

When looking into the 13 studies (17, 18, 22, 23, 25, 28, 31, 36, 37, 39, 42–44), a total of 802 ultrasound examinations provided complete diagnostic accuracy information, irrespective of stroke type and ultrasound modality. The bivariate diagnostic random-effects meta-analysis revealed an average sensitivity of 81.1% [95% CI (73.1, 87.2%)] and an average false positive rate of 14.5% [95% CI (6.7, 28.6%)], indicating that the diagnostic test has good accuracy. The AUC was 0.874, suggesting excellent diagnostic performance, with a partial AUC of 0.85 restricted to observed false positive rates. Between-study variability was moderate, with standard deviations of 0.597 for sensitivity and 1.453 for the false positive rate, while heterogeneity, as measured by I^2^, was low to moderate depending on the estimation approach. The Zhou and Dendukuri method estimated heterogeneity at 14.4%, and Holling’s adjusted methods reported very low heterogeneity (5.1–7.7%). These results demonstrate consistent diagnostic performance across studies, emphasizing the sensitivity, false positive rate, and AUC as the most interpretable indicators of accuracy. Figure 3 presents the HSROC curve including all studies.

### 3.2 Transcranial Doppler

From 26 articles, only nine mention Transcranial Doppler (TCD) with ages ranging between 18 and 96 years old; it is important to note that only one of these studies looked at hemorrhagic stroke (7, 17, 27, 33, 34, 38, 39, 46, 49). In the included studies, intracranial lesions were examined by a transtemporal and suboccipital approach with a 1.8–2.5-MHz multifrequency probe and by the transorbital approach, with a 7–7.5-MHz probe.

For ischemic stroke, only two articles contained enough information of diagnostic accuracy to conduct the meta-analysis (17, 39); due to the low number of studies, the meta-analyses could not be performed.

Regarding hemorrhagic stroke, both middle cerebral arteries were insonated through a temporal acoustic window to compare the unaffected MCA with the artery ipsilateral to the hematoma (34). Only one study assessed TCD in hemorrhagic stroke, in which 51 patients with ages from 43 to 78 were evaluated; however, it did not report sensitivity or specificity but rather the correlation between the pulsatility index as well as the systolic, diastolic, and mean velocities with hematoma volume, hypoattenuating volume and midline shift (34); which is why it was not included in our meta-analysis. They found that the pulsatility index of TCD was positively correlated with these variables (*p* < 0.05) and that it was significantly increased in patients with intraventricular hemorrhage (*p* = 0.094) (34).

#### 3.2.1 Diagnostic signs

Finally, when looking at the frequency of the diagnostic signs, studies regarding ischemic stroke reported a reduction in peak diastolic flow velocity in the middle cerebral artery (MCA) in 77% of patients, while in 9% focal stenosis and micro embolic signs were found by a transtemporal and suboccipital approach (7, 27, 33, 39, 49). High resistance or absent M-mode flow signatures in the proximal MCA were also present in 87% of MCA occlusions (40).

### 3.3 Transcranial color-coded ultrasound

**Figure 4:**
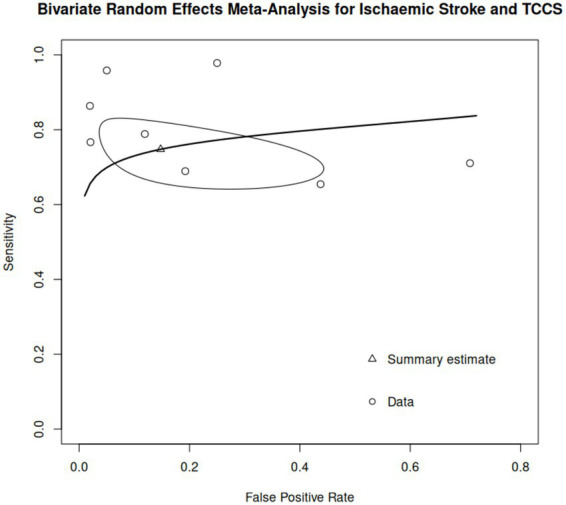
Hierarchical summary receiver operating characteristic (HSROC) curve for the analysis of TCCS for ischemic stroke.

Seventeen studies involved TCCS for the assessment of CVA (18, 22, 23, 25, 26, 28–32, 35–37, 42–45); of these, two studies provided information for both hemorrhagic and ischemic stroke (18, 44). For ischemic stroke, only 8 articles had sufficient information for our meta-analysis (22, 23, 25, 28, 36, 37, 43, 44), these studies included a total of 419 evaluations and reported a sensitivity ranging between 65 and 98% and a specificity ranging between 29 and 98% (Table 3). Using a bivariate diagnostic random-effects model estimated via REML, we found an overall sensitivity of 74.8% (95% CI, 66.4–81.6%) and a false positive rate of 14.8% (95% CI, 4.9–37%). The model’s diagnostic accuracy was robust, with an AUC of 0.796 and a partial AUC of 0.774. Variance across studies indicated low to moderate heterogeneity, with the Zhou and Dendukuri approach suggesting no heterogeneity (*I*^2^ = 0%), while adjusted Holling estimates indicated some heterogeneity (*I*^2^ = 6.5–11.1%). A HSROC can be found in Figure 4.

**Figure 5:**
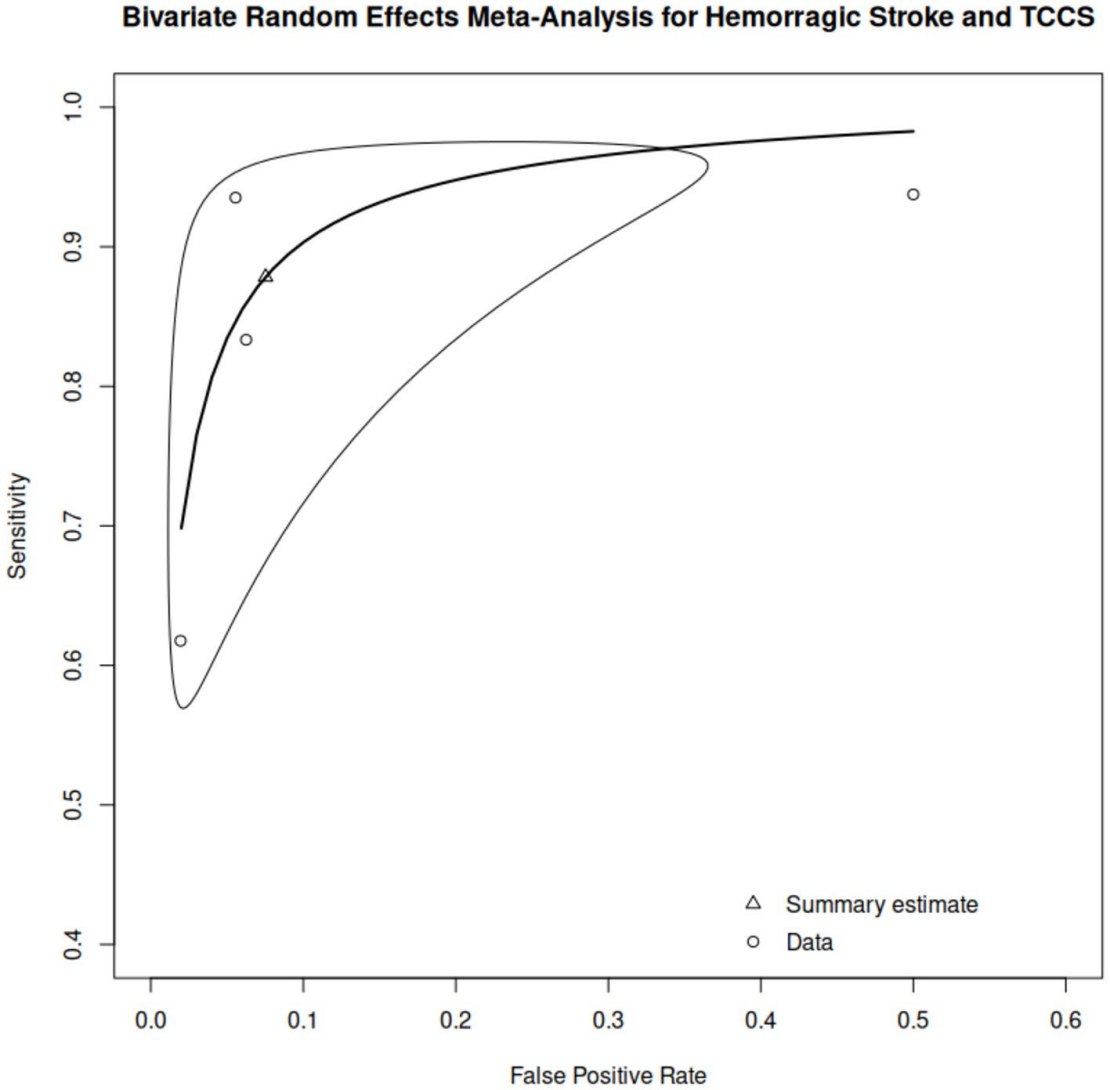
Hierarchical summary receiver operating characteristic (HSROC) curve of ultrasound for hemorrhagic stroke.

In contrast, five papers evaluated hemorrhagic stroke with 283 ultrasound examinations (18, 31, 32, 44, 45); of these, three articles provided enough diagnostic information for our analysis (18, 31, 44). In addition to these three articles, another study looking at hemorrhagic stroke with transcranial point-of-care ultrasound (POCUS) was also included in the meta-analysis (42). Their reported sensitivity ranged from 61.8 to 93.8% and specificity from 50.0 to 98.1% (Table 3). The results from the analyses yielded a high overall sensitivity of 87.8% (95% CI, 65.0–96.6%) and a low false positive rate of 7.5% (95% CI, 1.7–28.1%). Variance estimates demonstrated moderate between-study variability, with a standard deviation of 1.160 for sensitivity and 1.411 for the false positive rate, and a strong positive correlation (0.896) between them. The model achieved an excellent diagnostic accuracy, with an AUC of 0.953 and a partial AUC of 0.926. Heterogeneity was low according to the Zhou and Dendukuri approach (*I*^2^ = 13.1%) and remained moderate in the unadjusted Holling estimates (*I*^2^ = 30.2–49.9%), decreasing to very low levels with sample size adjustments (*I*^2^ = 3.1–3.9%). The resulting HSROC curve can be seen in Figure 5. Sensitivity analysis was not performed as we only included four articles in our analysis.

#### 3.3.1 Diagnostic signs

For ischemic stroke, multiple diagnostic signs were reported. Peak systolic flow and end diastolic flow velocities were amongst the most reported variables found in our study (18–20, 22–26, 28–32, 35–37, 43–45, 47, 48). There were also signs that portrayed significant cerebrovascular changes such as discontinuation of the color-coded signal in the middle cerebral artery (MCA) trunk, found by Postert in 47–100% of patients (36, 37). A German study detected the presence of thrombus in the MCA with hyperechoic B-mode patterns, reporting ischemia in 31% of their patients; additionally, they also reported hyperechoic linear patterns (45%) and decreases in flow velocity (25%) (30).

On the other hand, signs related with hemorrhagic stroke were related with hematoma detection or hemorrhagic transformation. A Japanese study detected the presence of cerebral hematomas in 100% of its participants with adequate bone windows (32). Hyperechoic intraventricular spaces or bleeding (68%), midline shift (91%) and ventricular growth (100%) could also be found in hemorrhagic stroke patients (31). In general, complications and hemorrhagic transformations could be detected in 83% of hemorrhagic stroke patients (with appropriate bone windows), with the signs listed above (18, 31, 32, 44, 45).

## 4 Discussion

Our systematic review and meta-analysis evaluated the diagnostic accuracy of transcranial ultrasonography, including transcranial Doppler (TCD) and transcranial color-coded duplex sonography (TCCS), in identifying ischemic and hemorrhagic stroke. Our findings demonstrate that transcranial ultrasonography has good diagnostic accuracy, with an overall sensitivity of 81.1% (95% CI [73.1, 87.2%]) and specificity of 85.5% (95% CI [71.4, 93.3%]) across 13 studies and 802 ultrasound examinations (17, 18, 22, 23, 25, 28, 31, 36, 37, 39, 42–44). The AUC of 0.874 further supports its diagnostic performance and potential utility in resource limited settings. Certainly, these results align with previous studies highlighting the utility of transcranial ultrasonography in stroke diagnosis, particularly in settings where advanced imaging modalities like MRI or CT are unavailable or delayed (51, 52). However, an important methodological consideration in interpreting our findings relates to the heterogeneity of ultrasonographic targets assessed across the included studies. Transcranial ultrasonography, particularly when applied in acute stroke settings, is not typically used to interrogate a single predefined ultrasonographic sign in isolation. Rather, diagnostic reasoning is based on the integration of multiple hemodynamic indicators, such as flow velocity asymmetries, absence or attenuation of signal, collateral patterns, or microembolic signals, and, in the case of transcranial color-coded duplex sonography, selected parenchymal or ventricular features suggestive of hemorrhage or mass effect. Consequently, most primary studies reported diagnostic accuracy metrics for an overall ultrasound-based diagnostic conclusion rather than for individual ultrasonographic abnormalities. For this reason, further stratification by specific ultrasonographic targets was not feasible in a systematic or methodologically robust manner. Importantly, attempting *post hoc* reclassification of studies according to presumed diagnostic constructs would have required subjective interpretation of outcomes not explicitly defined by the original authors, thereby introducing additional bias. Instead, we adopted *a priori* stratification by stroke subtype and ultrasound modality, which represent the most clinically and pathophysiologically meaningful axes of differentiation supported by the available data. This approach allowed us to preserve conceptual coherence while reflecting how transcranial ultrasonography is pragmatically applied in real-world stroke assessment.

Furthermore, while we observed low to moderate heterogeneity across studies, we decided not to conduct a formal meta-regression to explore potential sources of this variability. This decision was based on several methodological considerations. First, key covariates such as operator expertise, level of training, and proper and comparable ultrasound device specifications were either inconsistently reported or entirely absent in many of the included studies, limiting the feasibility of reliable covariate coding. Second, we addressed heterogeneity through subgroup analyses stratified by stroke type (ischemic vs. hemorrhagic) and ultrasound modality (TCD vs. TCCS), as well as through sensitivity analyses that excluded statistical outliers. Certainly, several studies exhibited outlier diagnostic values that diverged from the pooled estimates. For example, Sarwal et al. (42) reported a specificity of only 50%, likely due to the very small sample size (*n* = 11), which renders sensitivity and specificity estimates highly unstable. Furthermore, this study employed point-of-care ultrasound (POCUS), a modality with limited standardization and spatial resolution compared to conventional TCD or TCCS, which may reduce diagnostic precision. Similarly, Antipova et al. (18) reported a notably low sensitivity (24%) for ischemic stroke, while Madkour et al. (28) reported a specificity of just 29%. These outlier values reinforce the importance of methodological consistency, operator training reporting, and adequate sample sizes. They also underscore why we performed a sensitivity analysis excluding these outliers, which improved the model’s stability and diagnostic accuracy. We recommend that future studies adopt rigorous design standards, clearly report operator experience, and utilize validated protocols to ensure reproducibility and comparability of diagnostic estimates.

These methods allowed us to assess variability in a clinically meaningful manner while avoiding the risk of overfitting. Finally, the relatively limited number of studies included in the meta-analysis (*n* = 13) further constrained the statistical power for meta-regression and raised concerns about the stability of estimates if the dataset were subdivided further. We therefore focused on robust stratified analyses and transparent reporting rather than formal meta-regression, while highlighting the need for future studies to more consistently report potentially influential methodological factors.

**Figure 6:**
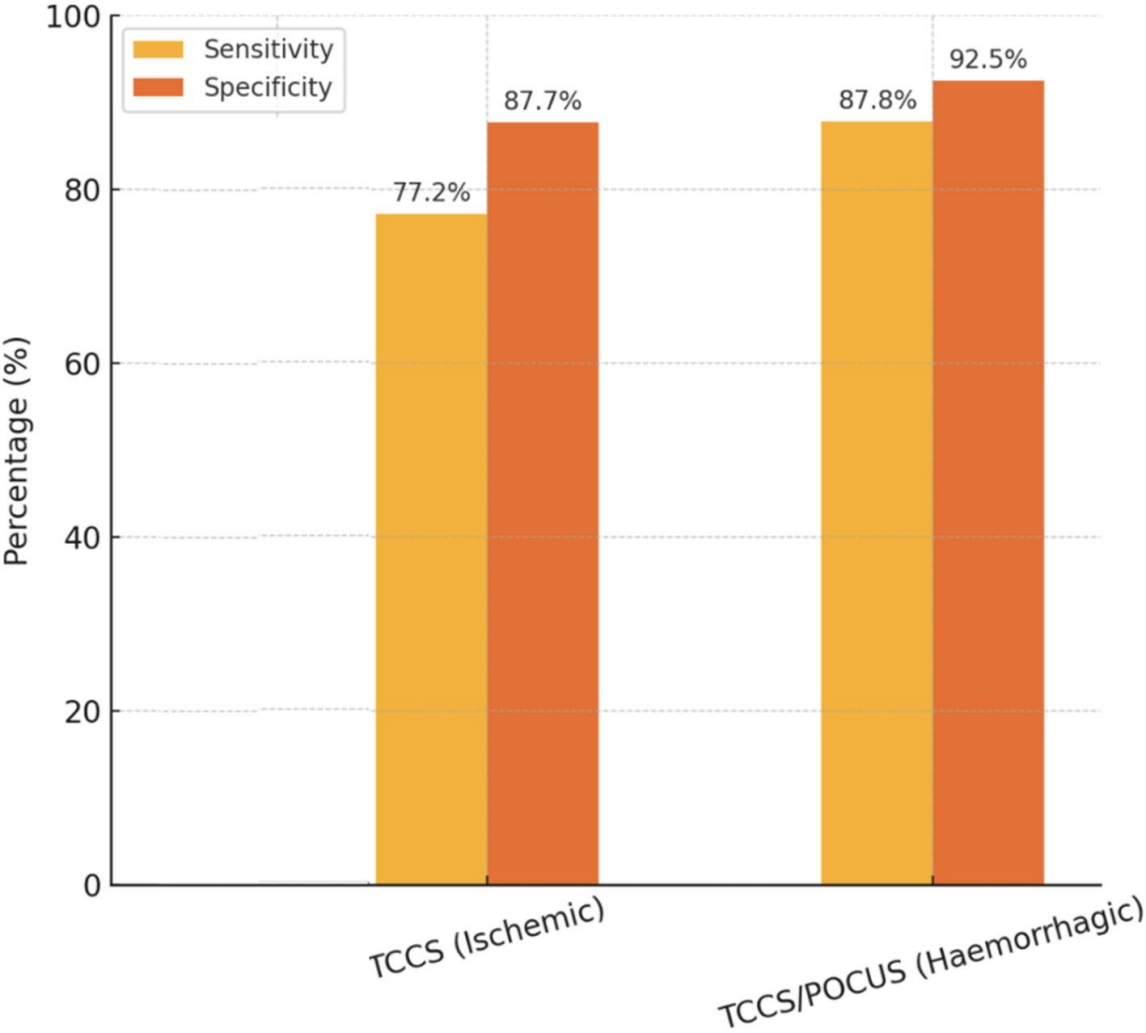
Diagnostic accuracy of transcranial ultrasonography by modality and stroke type.

When stratified by stroke type, TCCS showed higher sensitivity (87.8%) for hemorrhagic stroke compared to ischemic stroke (74.8%), while TCD could not be evaluated due to the scarcity of available evidence reporting the diagnostic accuracy information of this US modality. The superior performance of TCCS in hemorrhagic stroke may be attributed to its ability to visualize parenchymal changes, such as hematomas and midline shifts, which are critical diagnostic signs in hemorrhagic stroke (37, 53). Furthermore, the heterogeneity observed in diagnostic accuracy across studies, particularly for TCCS in ischemic stroke, underscores the importance of standardized protocols and operator expertise. Finally, studies with outlier results emphasize the need for rigorous methodological quality in future research and standardization of transcranial ultrasound (54). Figure 6 compares the diagnostic accuracy of the different modalities of transcranial ultrasonography in our meta-analysis.

Our review identified key diagnostic signs for both stroke types. For ischemic stroke, reductions in peak diastolic flow velocity and focal stenosis in the middle cerebral artery were commonly reported. In hemorrhagic stroke, hyperechoic intraventricular spaces, midline shifts, and ventricular growth were frequently observed. These findings highlight the potential of transcranial ultrasonography not only for initial stroke diagnosis but also for monitoring disease progression and complications; the utility of ultrasound signs in stroke and intracranial pathology has already been reported (55, 56). Moreover, transcranial ultrasonography holds significant promise for stroke diagnosis and management in resource-limited settings, where access to advanced neuroimaging modalities such as MRI and CT is often constrained by cost, infrastructure, and availability (57–59). In these settings, ultrasound offers a portable, cost-effective, and real-time imaging alternative that can be deployed at the bedside, in emergency departments, or even in prehospital settings (60).

Several studies have demonstrated the feasibility and impact of ultrasound in low-resource environments. For instance, in sub-Saharan Africa, where stroke burden is high and diagnostic resources are limited, TCD has been successfully used to screen for intracranial stenosis and monitor cerebral blood flow in stroke patients (61, 62). The portability of ultrasound devices also makes them ideal for use in mobile health units and remote clinics, enabling early diagnosis and triage of stroke patients who might otherwise face delays in receiving care. Furthermore, the relatively low cost of ultrasound equipment compared to MRI or CT scanners makes it a sustainable option for healthcare systems in low- and middle-income countries (LMICs) to be used in stroke units; stroke units offer an important benefit for patients even when they do not provide imaging modalities (63). Therefore, is TCD and TCCS can achieve important diagnostic accuracy if well-performed, more studies are needed regarding the application of transcranial ultrasonography in stroke-units in LMICs and the impact of such implementation, especially of TCD as we could not find data robust enough to be included in our meta-analysis. Moreover, the use of ultrasound in stroke care extends beyond diagnosis. It can also be used to monitor patients during treatment, assess the effectiveness of interventions, and detect complications such as hemorrhagic transformation or increased intracranial pressure (64). This real-time monitoring capability is particularly valuable in resource-limited settings, where patients may not have access to continuous advanced imaging. Despite its advantages, challenges exist in scaling up the use of transcranial ultrasonography in LMICs. These include the need for ongoing training, maintenance of equipment, and integration into existing healthcare systems (60, 65). Addressing these challenges, in particular proper training for healthcare providers in transcranial ultrasonography, will require coordinated efforts from governments, non-governmental organizations, and the global health community to ensure sustainable implementation.

Finally, when interpreted in the context of current clinical standards, the findings of this meta-analysis help clarify the potential, yet underdefined, role of transcranial ultrasonography within stroke care pathways. Contemporary international stroke guidelines consistently prioritize non-contrast computed tomography or magnetic resonance imaging as the reference standards for acute stroke diagnosis, while transcranial Doppler and transcranial color-coded duplex sonography are either minimally addressed or relegated to adjunctive roles, such as hemodynamic monitoring or research applications. This cautious positioning largely reflects the heterogeneity of the existing evidence base rather than definitive evidence of limited utility. Our results provide consolidated diagnostic accuracy estimates that help delineate where transcranial ultrasonography may add value, particularly by demonstrating higher sensitivity of TCCS for hemorrhagic stroke and clinically meaningful accuracy for ischemic stroke detection in selected contexts. These data support the consideration of transcranial ultrasonography as a complementary diagnostic tool in scenarios not fully addressed by current guidelines, such as prehospital triage, early bedside assessment when CT or MRI is delayed or completely, and stroke evaluation in low-resource settings. Importantly, our findings also underscore that any guideline expansion should be conditional on adequate operator training, standardized acquisition protocols, and explicit acknowledgment of the technique’s limitations, thereby providing a pragmatic framework for future guideline development rather than advocating indiscriminate adoption.

### 4.1 Future directions

Future research should focus on several key areas to further enhance the utility of transcranial ultrasonography in stroke care. First and foremost, there is a need for standardized protocols and guidelines to ensure consistency in the use and interpretation of ultrasound findings. This has been attempted in the past with the last guideline published in 2004 by the American Academy of Neurology (66); however, this guideline was retired on February 23, 2018, due to not been updated or reaffirmed in 5 years. This showcases the lack of focus in the use of transcranial ultrasound and its potential benefits as an accessible and cheap neuroimaging methodology. Transcranial ultrasound has even been used to create a classification for thrombolysis in brain ischemia for clinical trials, showing that such TCD classification correlated with stroke severity, mortality, and recovery after thrombolytic therapy (67). Standardization should encompass several key domains. Structured training programs and formal certification pathways should be developed for physicians, sonographers, and emergency personnel, with a focus on image acquisition, interpretation, and stroke-specific findings; these programs would be especially impactful in low-resource settings, where consistent operator training can mitigate variability and expand diagnostic capacity. Device calibration protocols must be implemented to ensure inter-machine consistency, including periodic performance verification and harmonization of frequency settings, gain levels, and probe usage. Additionally, beyond procedural standardization, specific diagnostic targets should be integrated into routine protocols; for instance, in hemorrhagic stroke, midline shift, ventricular enlargement, and intraventricular echogenicity should be routinely evaluated using TCCS, while in ischemic stroke, velocity-based occlusion grading in the MCA or ICA (e.g., using power M-mode or contrast enhancement) should be reported. The inclusion of such indicators would enhance reproducibility and clinical applicability, especially in triage scenarios.

Second, more studies are needed to evaluate the cost-effectiveness of ultrasound compared to traditional imaging modalities in different healthcare settings ad to properly validate its use. Certainly, to robustly validate transcranial ultrasound as a frontline neuroimaging modality, prospective multicenter trials are urgently needed. These studies should compare TCD and TCCS directly with CT, MRI, or angiography in the same patient cohorts, ideally across a range of hospital settings, including stroke units in low- and middle-income countries (LMICs). These trials should assess not only diagnostic performance metrics (e.g., sensitivity, specificity, and inter-rater reliability) but also logistical and economic outcomes such as time-to-diagnosis, feasibility of bedside use, and cost-effectiveness. Such data would help define the clinical niche of transcranial ultrasound and support its inclusion in regional or national stroke guidelines.

Finally, technological advancements in ultrasound equipment, such as the development of more portable and affordable devices, could further expand its use in resource-limited settings (68). Innovations in artificial intelligence and machine learning may also enhance the accuracy and reliability of ultrasound interpretations, reducing the reliance on operator expertise (69). AI offers promising tools to enhance image interpretation, automate flow velocity analyses, and assist novice operators. However, these technologies require careful validation in real-world clinical environments. Future studies should explore the integration of AI into ultrasound software platforms within prospective trial designs, particularly in LMICs where sonographer variability may limit diagnostic consistency. Together, these advancements could position transcranial ultrasonography as a cost-effective, scalable, and accurate neuroimaging alternative where traditional modalities are unavailable or delayed. We would like to highlight this last point as, by no means, are we suggesting TCD or TCCS as replacements of CT or MRI where available; however, many countries do not have the luxury of having CT or MRI readily available due to economical constraints and patients are suffering due to a lack of viable alternatives.

### 4.2 Limitations

Several limitations should be acknowledged. First, the exclusion of non-English and non-Spanish studies may have introduced language bias. Second, the variability in study designs, sample sizes, and diagnostic criteria across included studies may have influenced the pooled estimates. Third, the lack of data on operator experience and inter-rater reliability in many studies limits the generalizability of our findings (70). Third, the small number of studies evaluating hemorrhagic and ischemic stroke with TCD precluded a comprehensive meta-analysis for this subgroup. Fourth an important limitation of this review is that several included studies reported diagnostic accuracy based on the number of ultrasound examinations rather than the number of individual participants. In many cases, a single patient underwent multiple assessments targeting different intracranial structures, and the reported data did not allow us to disaggregate findings at the participant level. As a result, sensitivity and specificity were calculated using the number of ultrasound examinations as the unit of analysis, in line with the approach taken by the original studies. While this method preserves the integrity of the reported accuracy metrics, it may slightly overrepresent the precision of pooled estimates by inflating the denominator. Finally, the diagnostic constructs evaluated across included studies were heterogeneous, encompassing a broad range of hemodynamic and, to a lesser extent, structural ultrasonographic findings. Although this heterogeneity reflects real-world ultrasound practice, it limits the interpretability of pooled diagnostic accuracy estimates as representing a single, uniform diagnostic test. Due to inconsistent reporting across studies, we were unable to stratify analyses according to specific ultrasonographic abnormalities, such as flow acceleration, microembolic signals, absence of detectable flow, or parenchymal echogenic changes; most studies did not provide separate sensitivity and specificity estimates for individual ultrasound features, precluding reliable disaggregation. As a result, summary estimates should be interpreted as aggregate indicators of diagnostic performance within defined modality and stroke subtype strata rather than as definitive measures of any single ultrasonographic sign. Additionally, variability in operator expertise, equipment quality, insonation windows, and clinical context likely contributed to residual heterogeneity despite the use of bivariate random-effects models. Future diagnostic studies would benefit from standardized reporting frameworks that explicitly link individual ultrasonographic findings to diagnostic accuracy outcomes, thereby enabling more granular and mechanistically informative evidence synthesis.

## 5 Conclusion

Transcranial ultrasonography, particularly TCCS, demonstrates good to excellent diagnostic accuracy for both ischemic and hemorrhagic stroke. Its non-invasive nature, portability, and real-time imaging capabilities make it a valuable tool in stroke diagnosis, especially in resource-limited settings. Future research should focus on standardizing protocols, improving operator training, and expanding studies to underrepresented regions to further validate its clinical utility. Incorporating transcranial ultrasonography into stroke diagnostic algorithms could enhance early detection and improve patient outcomes globally. However, these findings should be interpreted with caution, as only a minority of included studies were classified as having low risk of bias, while a substantial proportion exhibited moderate or high methodological limitations. Consequently, the reported diagnostic accuracy estimates should be regarded as indicative rather than definitive, underscoring the need for higher-quality, prospectively designed studies with standardized acquisition protocols and transparent reporting to more robustly define the role of transcranial ultrasonography in stroke diagnosis.

## Data Availability

The original contributions presented in the study are included in the article/supplementary material, further inquiries can be directed to the corresponding author.
